# Chemical Analysis of SU-Eohyeol Pharmacopuncture and Its *In Vitro* Biological Activities

**DOI:** 10.7150/ijms.100083

**Published:** 2024-10-03

**Authors:** Sarah Shin, No Soo Kim

**Affiliations:** 1 KM Science Research Division, Korea Institute of Oriental Medicine, Daejeon 34054, Republic of Korea.; 2 KM Convergence Research Division, Korea Institute of Oriental Medicine, Daejeon 34054, Republic of Korea.; The authors contributed equally to this work.

**Keywords:** pharmacopuncture, gas chromatography-mass, liquid chromatography-mass, BV2 microglial cells, anti-inflammatory, antioxidant

## Abstract

**Introduction:** Pharmacopuncture (PA) is widely used in traditional Korean medicine to treat various diseases, including abdominal obesity, nervous system diseases, and musculoskeletal disorders. In the present study, we attempted to identify the chemical components of SU-Eohyeol PA (SUEHP), comprising extracts of eight medicinal herbs and Cervi Parvum Cornu, using gas chromatography-mass spectrometry (GC-MS) and liquid chromatography-mass spectrometry (LC-MS) and evaluated the* in vitro* anti-inflammatory and antioxidant activities of SUEHP.

**Methods:** Volatile components of SUEHP were identified by GC-MS analysis of the n-hexane, dichloromethane (DCM), and distilled water-acetonitrile (DW-CAN) solvent fractions. LC-MS was performed to identify small metabolites of SUEHP using a water-methanol solvent fraction. *In vitro* anti-inflammatory and antioxidant potential of SUEHP was evaluated using cell-free biochemical assays and a BV2 microglial cell culture system.

**Results:** GC-MS of SUEHP using the n-hexane, DCM, and DW-ACN solvent fractions detected 32 components. The major components (>3%) were 9-octadecenamide (25.9%) and eicosane (5.4%) in n-hexane fraction, 9-octadecenamide (29.1%) and cis-11-octadecenoic acid (3.1%) in DCM fraction, and 9-octadecenamide (31.1%) and 9-octadecenoic acid (17.9%) in DW-CAN fraction. LC-MS of SUEHP using a water-methanol solvent fraction detected 36 primary metabolites, including (a)symmetric dimethylarginine, *L*-pyroglutamic acid, *n*,*n*-dimethylglycine, *n*-acetyl-L-aspartic acid, and amino acids. Enrichment analysis and subsequent network analysis of the primary metabolites suggested their association with neurodegenerative diseases, including Alzheimer's disease and schizophrenia. Cell-free biochemical assays and molecular signaling studies of lipopolysaccharide-stimulated BV2 murine microglial cells demonstrated the anti-inflammatory and antioxidant activities of SUEHP.

**Conclusion:** The present study identified the biochemically active components of SUEHP and suggested their therapeutic potential against diseases related to inflammatory and oxidative stress.

## Introduction

Pharmacopuncture (PA), a unique acupuncture therapy injecting herbal extract solution on the acupoints, has been increasingly practiced in a traditional Korean medicine (TKM)^1^, ^2^. The herbal medicine used for PA is prescribed according to the theory of “Science of Prescription (Bang-Je-Hak in TKM)^3^ and crude extract solutions of single or multiple herbal medicine undergo further purification and dilution process before injection. Therefore, PA combines herbal medicine with acupuncture expecting their therapeutic synergism by integrating physical stimulation of acupuncture and pharmaco-chemical effects of herbal medicine^4^, ^5^. In local clinics, PA has been used to treat or improve diverse diseases including abdominal obesity, nervous system diseases, and most frequently musculoskeletal disorders^2^, ^5^. Because PA does not need the absorption process through the gastrointestinal tract and injects the herbal extract on acupoint according to the meridian system, an immediate pharmacological effect can be expected. Another advantage of PA is that it can accurately control the drug dosage and can also be used in patients with reduced eating and digestive function^6^.

PA of Eight Principles (Pal-Gang PA in Korean), one of PA categories, is prescribed based on a TKM diagnostic method: “Pal-Gang” syndrome differentiation of yin-yang, exterior-interior, cold-heat, and deficiency-excess^7^. Jungsongouhyul PA that is included in PA of Eight Principles is used for “extravasated blood or blood stasis (“Ou-Hyul” syndrome differentiation)”^4^. The herbal medicine of Jungsongouhyul PA is composed of Gardeniae Fructus, Olibanum, Myrrha, Corydalis Tuber, Persicae Semen, Salviae Miltiorrhizae Radix, Paeoniae Radix, Sappan Lignum^4^. In experimental animal models, Jungsongouhyul PA was reported to be efficient for pain relief and nerve regeneration after sciatic nerve injury^8^, ^9^, fracture healing during early stage^10^, and neuroprotection in traumatic brain injury^11^. Over the past several decades, the clinical studies that were primarily conducted by TKM clinicians have shown that Jungsongouhyul PA was helpful for central post stroke pain^12^, low back pain^13^, acute traumatic shoulder pain^14^, radial nerve palsy^15^, and Parkinson's disease (PD)-associated chest pain^16^. Recently, Jungsongouhyul PA was further modified to SU-Eohyeol PA (SUEHP) by supplementing Jungsongouhyul PA with a refined extract of Cervi Parvum Cornu^1^. The extract of Cervi Parvum Cornu was known to exert anti-inflammatory, anti-stress and anti-aging properties, and it has been used in TKM for maintenance of hemostasis, improvement of immunity and liver function, growth promotion, and supplement of essential body fluids in human tissues^1^, ^17^. Clinical application of SUEHP is expected to be 0.1-1.0 ml/human (0.1 ml/time)^1^.

A previous comparative clinical study showed that combination of SUEHP with conservative TKM treatment involving acupuncture, herbal medicine, cupping, and physical therapy was more efficient to improve low back pain when compared with conservative TKM treatment only^18^. Another clinical case study showed that SUEHP could improve the motor coordination function of PD patients^19^. Since then, few clinical studies were done to evaluate the efficacy in other diseases or clinical symptoms. Recently, a pilot clinical study was initiated to evaluate the efficacy of SUEHP to improve the pain experienced by PD patients^4^. Meanwhile, several pre-clinical toxicity studies with SUEHP showed that the lethal dose of intramuscular injection of SUEHP exceeded 1.0 ml in experimental rats^1^ and no significant genotoxicity was observed in both the micronucleus test using the rat bone marrow cells^20^ and the chromosome aberration test using the Chinese hamster lung cells^21^. However, to date, information on the chemical composition and biological activity of SUEHP is very limited. In the present study, we analyzed the chemical compositions of SUEHP using a gas chromatography-mass spectrometry (GC-MS) and a liquid chromatography-mass spectrometry (LC-MS) and evaluated its antioxidant and anti-inflammatory activities using both cell-free and BV2 murine microglial cell culture systems.

## Materials and methods

### SUEHP and chemicals

SUEHP (lot # NK0721003) was manufactured in accordance with the Korean Good Manufacturing Practice at External Herbal Dispensary of Namsangcheon Korean Medicine Clinic (Yongin, Republic of Korea). The detailed processes of SUEHP production has been previously described 1. The prescriptions for SUEHP are as follows: Cervi Parvum Cornu (*Cervus elaphus* Linné, 50 mg/ml), Gardeniae Fructus (*Gardenia jasminoides* Ellis, 75 mg/ml), Olibanum (*Boswellia carterii* Birdwood, 30 mg/ml), Myrrha (*Commiphora myrrha* Engler, 30 mg/ml), Corydalis Tuber (*Corydalis ternata* Nakai, 30 mg/ml), Persicae Semen (*Prunus persica* Batsch, 22.5 mg/ml), Salviae Miltiorrhizae Radix (*Salvia miltiorrhiza* Bunge, 22.5 mg/ml), Paeoniae Radix (*Paeonia lactiflora* Pallas, 22.5 mg/ml), Sappan Lignum (*Caesalpinia sappan* Linné, 22.5 mg/ml). SUEHP was supplied as a glass vial containing sterilized 3 ml liquid and stored in the dark at 4°C until used. All chemicals or solvents except those mentioned elsewhere were purchased from the Sigma-Aldrich (St. Louis, MO, USA).

### Chemical analysis of SUEHP by GC-MS

SUEHP solution was vigorously extracted for 10 min by adding equal volumes (5 ml) of three different solvents with different polarities and solubilities: n-hexane, dichloromethane (DCM), and a 1:1 mix of deionized water-acetonitrile (DW-ACN). The extraction was performed once, after which a solvent was collected from each extraction. The extract solution was centrifuged, and the supernatant was filtered through a 0.2 μm hydrophobic syringe filter. The filtered extract was then injected into GC-MS. SUEHP was qualitatively analyzed using the SHIMADZU GC-MS system (Shimadzu, Tokyo, Japan) consisting of a GC module (SHIMADZU GC-2010 Plus) and an MS module (SHIMADZU GCMS-QP2010 Ultra). The system utilized a DB-5MS capillary column (30 m × 0.25 mm i.d., with a film thickness of 0.25 μm) for chromatographic separation. The temperature in the GC oven was programmed to start at 40°C and hold for 2 minutes, then to a final temperature of 300°C for 10 min via ramp of 10°C/min. This method ensured the gradual volatilization and separation of compounds depending on their volatility and polarity. The injector was heated to 280°C, enabling rapid vaporization of the sample while preventing degradation. Ultrahigh-purity helium was used as the carrier gas at a constant flow rate of 1 ml/min, maintaining the column pressure under optimal conditions for separation. The split injection mode was used at a ratio of 20:1, and a sample volume of 1 µl was introduced into the column. The ion source operated by electron impact ionization at 70 eV was heated to 230°C, which promoted consistent ionization of analytes. The mass spectrometry data was acquired in a full scan mode from m/z 40 to 650, which facilitated detection of a broad spectrum of molecules. The interface temperature was maintained at 280°C and the system was tuned to detect ions effectively over the entire mass range. The naked peak features were identified by comparing retention times and mass spectra with those in the NIST 14 and Willy 7 Mass Spectral Libraries to determine their chemical structures, which were further confirmed by comparing them with previously published literature.

### Chemical analysis of SUEHP by LC-MS

SUEHP solution (500 μl) was vacuum-dried, and the residual pellet was vigorously extracted with 1 ml of a cold water-methanol (1:1, vol/vol) mixture for 20 min in an ultra-sonicator and then centrifuged at 6500 rpm for 10 min at 25°C. The supernatant was analyzed as an analyte. Instrumental conditions for the analysis were used as a previously described method 22. Briefly, the primary metabolites composition of SUEHP was analyzed simultaneously using ultra-high performance liquid chromatography (UHPLC, 1290 infinity Ⅱ LC system, Agilent Technologies, Santa Clara, CA, USA) coupled to a quadrupole time-of-flight mass spectrometry (Q-TOF-MS, 6546 Q-TOF, Agilent Technologies system). The mobile phase for positive mode consisted of 10 mM ammonium formate containing 0.1% formic acid in water (solvent A) and 10 mM ammonium formate in an acetonitrile-water mixture (9:1, vol/vol; solvent B) containing 0.1% formic acid. The flow rate was set to 0.3 ml/min, and the temperature was maintained at 45°C for a total run time of 23 min. The linear gradient of UHPLC was as follows: 90% B for 3 min, 90% B to 35% B from 1 min to 12 min, 35% to 25% B from 12 min to 12.5 min, 25% to 90% B from 12.5 min to 14.5 min, and then equilibrated until 23 min. The mass spectrometer was operated in positive and negative ionization modes with a mass range of m/z 50 to 1700. MS parameters were set as follows: gas temperature 225°C, drying gas 13 l/min, nebulizer 35 psi, fragmentor 125 V, skimmer 45 V, and capillary voltage 3500 V for a positive ion mode; gas temperature 325°C, drying gas 6 l/min, nebulizer 45 psi, fragmentor 140 V, skimmer 65 V, and capillary voltage 3500 V for a negative ion mode. A narrow isolation window (1.3 Da) was applied to acquire MS/MS data at collision energies of 20 V. UHPLC/Q-TOF-MS data obtained for non-targeted metabolic profiling were processed with MS-Dial (v4.90, http://prime. psc.riken.jp/) to detect features, perform alignment, and generate peak tables of m/z and retention times in the samples. Naked peaks were putatively identified by matching retention time, m/z, and the ms/ms fragmentation information using the *in silico*-based metabolite spectrum databases, including MassBank of North America, GNPS, Fiehn HILIC, and the HMDB. Cut-off thresholder of metabolite identification score of 80% was calculated using MS1 similarity, ms/ms fragmentation similarity, and isotope ratio similarity 23.

### Biological pathway analysis of SUEHP

Integrated pathway analysis was performed using MetaboAnalyst 6.0 to elucidate the biochemical markers and diseases affected by SUEHP components. Enrichment analysis was initially performed to identify pathways significantly affected by differentially expressed metabolites. Then, network analysis was performed to map these metabolites to corresponding disease states using the relational database of known metabolite-disease interactions.

### Cell culture

A BV2 murine microglial cells, a kind gift from Dr. M.Y. Lee (Korea Institute of Oriental Medicine), were maintained in a 100 mm cell culture dish containing a growth medium consisting of Dulbecco's modified Eagle's medium with 25 mM glucose (#11995) supplemented with 10% (vol/vol) heat-inactivated fetal bovine serum (FBS, #16000044) and 1% (vol/vol) penicillin/streptomycin mix (#15140122) in a 5% CO_2_-balanced humidified incubator at 37°C. Cells were split in fresh growth medium every 2-3 days. All basal medium, serum, supplements, phosphate-buffered saline (PBS, pH7.4, #10010023), and cell culture plates/dishes were obtained from the Thermo Fisher Scientific (Thermo Fisher Scientific, Rockford, IL, USA).

### Cell viability assay

The effects of SUEHP on BV2 cell viability was determined using the Quanti-Max^TM^ WST-8 cell viability assay kit (QM1000, Biomax, Guri, Republic of Kore) according to the manufacturer's instruction. Briefly, cells were plated at 1x 10^4^ cells per well in a 96-well cell culture plate. After 24 h, the cells were exposed to 0-20% (vol/vol) SUEHP and incubated for an additional 24 h. The cells were then washed once with PBS and incubated in fresh growth medium containing 10% (vol/vol) WST-8 solution. Color development was monitored using the SpectraMax3 Microplate Reader (Molecular Devices, Sunnyvale, CA, USA), and the relative cell viability of SUEHP-treated cells was determined by comparing the OD450 with that of PBS control-treated cells.

### Subcellular fractionation

BV2 cells were plated at 2.5 x 10^5^ cells per well in a 6-well cell culture plate and incubated for 24 h. The cells were pretreated with SUEHP (0-10%) for 2 h and then exposed to 100 ng/ml lipopolysaccharide (LPS, L2654) for an additional 2 h. The cells were then washed twice with ice-cold PBS and resuspended in 0.5 ml low salt buffer consisting of 10 mM HEPES (pH7.5, IBS-BH004, iNtRON Biotechnology, Seongnam, Republic of Korea), 10 mM KCl, 0.1 mM ethylene-diamine-tetraacetic acid (EDTA), and 1% (vol/vol) Halt^TM^ protease and phosphatase inhibitor cocktail (#78444, Thermo Fisher Scientific). Immediately after the addition of 25 μl of 10% (vol/vol) NP-40, the cell suspension was vigorously voltexed for 30 sec. After centrifugation at 3,000 rpm for 5 min at 4°C, the supernatant was transferred to a new 1.5 ml tube (cytoplasmic fractions). The nuclear pellet was washed once with 0.5 ml low salt buffer and resuspended in 50 μl high salt buffer consisting of 20 mM HEPES, 400 mM NaCl, 1 mM EDTA, and 1% Halt^TM^ protease and phosphatase inhibitor cocktail. After incubation for 45 min on ice with periodically vigorous voltex every 5 min, the nuclear extract was obtained by centrifugation at 12,000 rpm for 5 min at 4°C. Protein concentrations of cytoplasmic and nuclear extracts were determined using a bicinchoninic acid assay (BCA, #23227, Thermo Fisher Scientific).

### Nuclear factor kappa-light-chain-enhancer of activated B cells (NFκB) and nuclear factor erythroid 2-related factor 2 (Nrf2) transcription factor (TA) assays

The effects of SUEHP on NFκB or Nrf2 signaling in LPS-stimulated BV2 cells was determined using the Trans AM^TM^ NFκB p65 (#40096) and Nrf2 (#50296) TA kits (Active Motif, Carlsbad, CA, USA), respectively. Briefly, BV2 cells were plated at 5 x 10^5^ cells per well in a 60 mm cell culture dish and incubated for 24 h. The cells were pretreated with SUEHP (0-10%) for 2 h and then exposed to 100 ng/ml LPS for an additional 15 min. Nuclear extracts were prepared according to the manufacturer's instructions. Protein concentration in nuclear extracts was determined using the BCA method. Nuclear NFκB p65 or Nrf2 TF assays were performed using 10 μg of nuclear extracts, and color development was monitored using the SpectraMax3 microplate reader. The relative levels of nuclear NFκB p65 and Nrf2 TAs of cells treated with a combination of SUEHP and LPS were determined by comparing with those of PBS control-treated cells.

### Free radical scavenging assay

The cationic and anionic free radical scavenging activity of SUEHP were determined using cell-free 2,2′-azino-bis[3-ethylbenzothiazoline-6-sulfonic acid] (ABTS) and 1,1-diphenyl-2-picrylhydrazyl (DPPH) assays, respectively, as previously described 24 with slight modifications. Briefly, ABTS radicals were formed by mixing equal volumes of 7.4 mM ABTS (A2166, Tokyo Chemical Industry, Tokyo, Japan) with 2.6 mM potassium persulfate (K_2_S_2_O_8_) in the dark for 2 h. DPPH (#14805, Cayman Chemical, Ann Arbor, MI, USA) was dissolved at 0.2 mM in absolute methanol and kept in the dark until used. In a 96-well assay plate (Thermo Fisher Scientific), equal volumes of SUEHP serially diluted with PBS were mixed with ABTS or DPPH working solutions and incubated in the dark for 7 min (ABTS) or 30 min (DPPH) at 22-24°C. Ascorbic acid was also included in assays serving as a positive control. Bleaching by SUEHP was measured optically at 734 nm (ABTS) or 514 nm (DPPH) using the SpectraMax i3 (Molecular Devices) microplate reader. Free radical scavenging activity by SUEHP was determined using the following formula: inhibition (%) = (1-OD of SUEHP/OD of control buffer) x 100. Half maximal Inhibitory concentrations of SUEHP in ABTS and DPPH assays were calculated by a 4-parameter logistic function using the SigmaPlot program (ver. 14.5, Grafiti, Palo Alto, CA, USA).

### Nitric oxide (NO) production assay

The effect of SUEHP on NO production from LPS-stimulated BV2 cells in the presence and absence of SUEHP pretreatment was determined using the Griess reagent system (G2930, Promega, Madison, WI, USA). Briefly, BV2 cells were plated at 2.5 x 10^5^ cells per well in a 6-well cell culture plate, and incubated for 24 h. The cells were pretreated with SUEHP (0-10%) for 2 h and then exposed to 100 ng/ml LPS for an additional 24 h. The culture medium was transferred to a 1.5 ml tubes and centrifuged at 12,000 rpm for 1 min at 22-24°C to remove cell debris. The clear supernatant was applied to determine NO levels using the kit according to the manufacturer's instruction. Cell-free culture medium was used as a negative control. The color development was determined at 540 nm using the SpectraMax i3 microplate reader.

### Quantitative polymerase chain reaction (qPCR)

The changes in the intracellular mRNA levels of the target genes was determined using qPCR. Briefly, BV2 cells were plated at 2.5 x 10^5^ cells per well in a 6-well cell culture plate and incubated for 24 h. The cells were then pretreated with SUEHP (0-10%) for 2 h and then exposed to 100 ng/ml LPS. After 2 h, total RNAs were isolated from the cells using the Easy-Spin^TM^ total RNA extraction kit (#17221, iNtRON Biotechnology), and the concentration was determined using the NanoDrop 2000 spectrophotometer (Thermo Fisher Scientific). Synthesis of first strand cDNA was performed from 1 μg of total RNA using the High-capacity cDNA reverse transcription kit (#4368814, Thermo Fisher Scientific) according to the manufacturer's instruction. The qPCR was then performed in a 10 μl reaction mixture containing a 250 μM gene-specific primer pair (Genotech, Daejeon, Republic of Korea), 50-fold diluted first strand cDNA, and 1x Power SYBR^TM^ green PCR master mix (#4367659, Thermo Fisher Scientific) in the CFX96^TM^ real-time PCR system (Bio-Rad, Hercules, CA, USA). Target gene expression was normalized by the glyceraldehyde 3-phosphate dehydrogenase (*Gapdh*) housekeeping gene, and the relative expression of target genes comparing with PBS control-treated cells was calculated by applying the 2-^ΔΔCt^ method. Primer sequences of interleukin 1 beta (*Il1b*), nitric oxide synthase 2 (*Nos2*), prostaglandin-endoperoxide synthase 2 (*Ptgs2*), tumor necrosis factor (*Tnf*), and *Gapdh* are summarized in **Table 1**.

### Western blotting

Whole cell lysates were prepared from BV2 cells in RIPA buffer (#89901) containing 1% (vol/vol) Halt^TM^ protease and phosphatase inhibitor cocktail. Protein concentration was determined using the BCA method. Equal amounts of protein (20 μg per lane) were separated on the 4-12% Bolt^TM^ Bis-Tris plus mini protein gel (#NW04120BOX, Thermo Fisher Scientific) and electrically transferred to 0.22 μm nitrocellulose membranes (#1620112, Bio-Rad) for 1 h at 100 volts. The membrane was blocked with the EzBlock Chemi solution (AE-1475, ATTO, Daejeon, Republic of Korea) for 1 h at 22-24°C and then incubated overnight at 4°C in the blocking solution containing a primary antibody. After washing 3 times for 10 min with Tris-buffered saline containing 0.1% Tween 20 (TBS-T, IBS-BT008a, iNtRON Biotechnology), the membranes were incubated for 1 h at 22-24°C in the blocking solution containing an appropriate horseradish peroxidase (HRP)-conjugated secondary antibody. After washing 3 times for 10 min with TBS-T, the protein of interest was visualized using the Super-signal west pico plus chemiluminescent substrate (Thermo Fisher Scientific) and the Fusion SL imaging system (Vilber, Collégien, France). Band intensity was determined using the ImageJ software (NIH, Bethesda, MA, USA). β-actin was used as a loading control. The primary and HRP-conjugated secondary antibodies used for western blotting were as follows: β-actin (A1978, Sigma-Aldrich), heme oxygenase-1 (HO-1, Ab13248, Abcam, Cambridge, UK), cAMP response element-binding protein (CREB, #9197), nuclear factor of kappa light polypeptide gene enhancer in B-cell inhibitor alpha (IκBα, #9242), phosphor-IκBα(p-IκBα, serine 32, #2859), manganese superoxide dismutase (MnSOD, #13141), Nrf2 (#12721, Cell Signaling Technology, Beverly, MA, USA), HRP-conjugated mouse anti-rabbit IgG (sc-2357), HRP-conjugated mouse IgG kappa binding protein (sc-516102, Santa Cruz Biotechnology, Dallas, TX, USA).

### Pan-biochemical screening assay

Biochemical activity screening of SUEHP was performed by Eurofins Discovery (study no. TW04-0011871, assay no. PP223, Taipei, Taiwan). The screening panel consisted of 87 biochemical assays, including G-protein-coupled receptors, ionic channels, enzymes, transporters, and nuclear receptors. The tests were performed in duplicate at a fixed dose (1%, vol/vol) of SUEHP. Reference standards of each assay were run in parallel to ensure the validity of the results. The response was determined to be significant when SUEHP showed >50% inhibition or stimulation.

### Cyclooxygenase (COX) inhibition assay

The inhibitory activities of SUEHP against two COX isozymes, ovine COX-1 and recombinant human COX-2, were determined using the COX fluorescent inhibitor screening assay kit (#700100, Cayman, Ann Arbor, MI, USA) according to the manufacture's instruction. Briefly, SUEHP serially diluted with 0.9% saline was mixed and incubated for 5 min at 22-24°C in assay buffer containing hemin and COX enzymes. Enzyme reactions were then initiated by adding 1-acetyl-3,7-dihydroxyphenoxazine and arachidonic acid. After 2 min of incubation at 22-24°C, the fluorescence of resorufin, a by-product of COX enzyme activity, was read at excitation 540 nm and emission 595 nm using the SpectraMax i3 microplate reader. SC-560 (66 μM) and DuP-697 (60 μM) were included in reactions as COX-1 and COX-2 inhibitor controls, respectively. Enzyme inhibition was calculated using the following formula: % inhibition = (1-sample activity/initial activity) x 100 (%).

### Statistical analysis

Data analysis was performed using the GraphPad Prism (v7.05, GraphPad Software, San Diego, CA, USA). All data are presented as means ± standard deviation (S.D.). Means were compared between groups using one-way ANOVA and Dunnett's *post hoc* multiple comparison test. The mean difference was considered statistically significant at p<0.05.

## Results

### Chemical composition analysis of SUEHP by GC-MS

**Figure 1:**
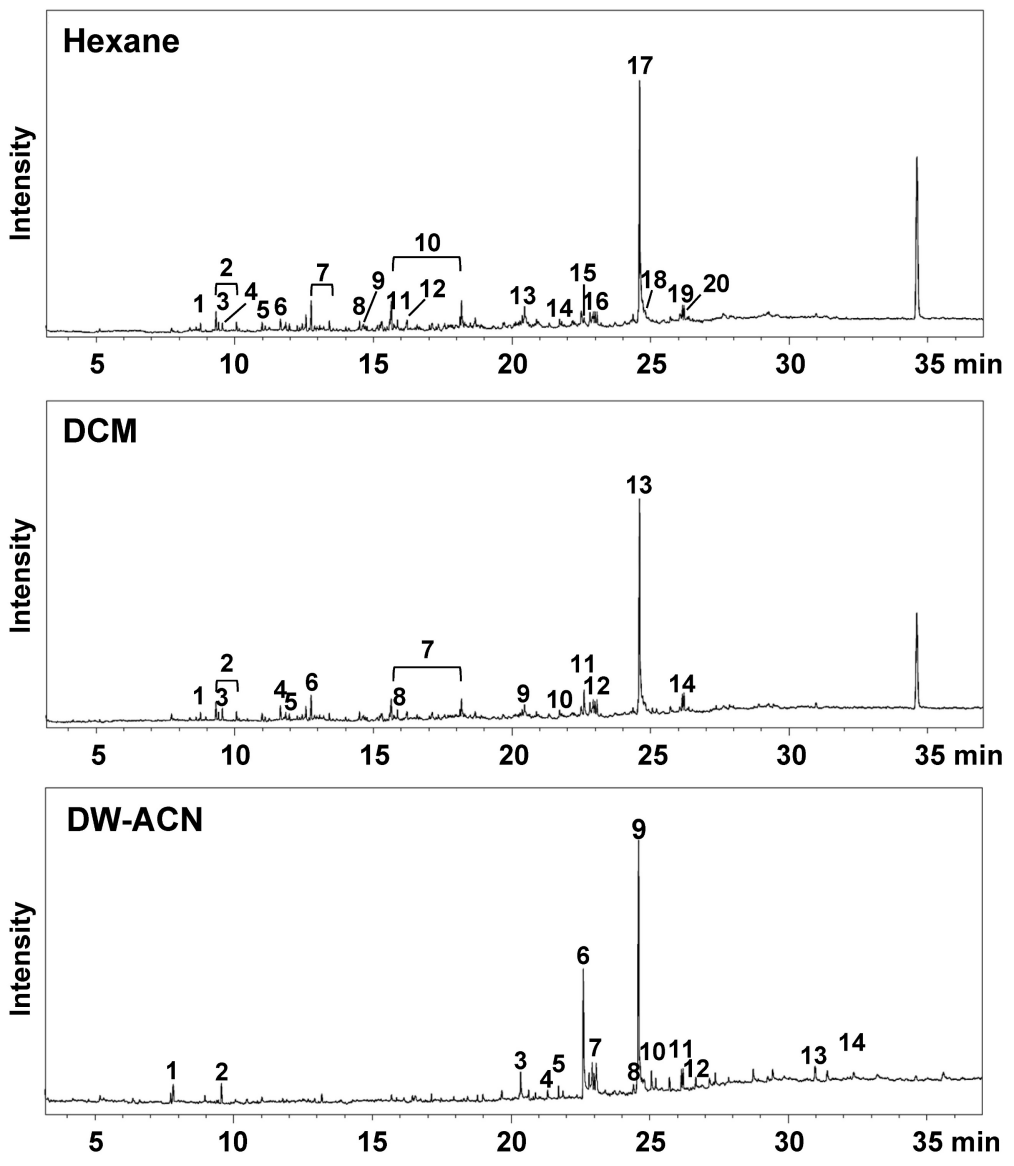
Total ion chromatograms of SUEHP extracted using n-hexane (upper trace), dichloromethane (DCM, middle trace), or deionized water-acetonitrile mixture (DW-ACN, lower trace).

The qualitative analysis of SUEHP was conducted using three solvents with differing polarities: n-hexane, DCM, and DW-ACN mixture. The resulting chromatograms are presented in **Fig. 1**. The chromatographic profiles of the extracts did not exhibit substantial variance in the overarching pattern contingent on the solvent used; however, differential peak profiles were discernible for certain analytes. For the n-hexane extract (upper trace in **Fig. 1**.), a total of twenty peaks were detected, with the major compounds identified as 9-octadecenamide (25.9%) and eicosane (5.4%) among others (**Table 2**). Notably, compounds such as 2-ethyl 2-methyl tridecanol, 3,8-dimethyldecane, 3,8-dimethylundecane, 4-methyldecane, 5-methyltetradecane, 8-methylheptadecane, dodecanamide, and tetradecane were uniquely extracted with n-hexane, emphasizing the importance of non-polar solvent selection for these analytes. In the DCM extract (middle trace in **Fig. 1**. and **Table 2**), 14 major peaks were discerned, with major compounds including 9-octadecenamide (29.1%) and *cis*-11-octadecenoic acid (3.1%). Interestingly, DCM proved effective in the extraction of medium-polarity compounds. The DW-ACN mixture extract (lower trace in **Fig. 1** and **Table 2**) exhibited a diverse array of fifteen compounds, highlighted by the presence of 9-octadecenamide (31.07%) and 9-Octadecenoic acid (17.92%). The polar nature of the DW-ACN mixture was conducive to the extraction of compounds such as 5-ethyl-2-methylheptane, which were not as prominent in the non-polar extracts. Moreover, 9-octadecenamide was a major peak observed across all extracts, accounting for 26% of the n-hexane extract, 29% of the DCM extract, and 31% of the DW-ACN extract, which indicates a significant portion in each chromatogram. Conversely, *cis*-11-octadecenoic acid consistently appeared as a minor peak in all extracts, demonstrating the selective extraction effects of solvents with varying polarities.

### Chemical composition analysis of SUEHP by LC-MS

**Figure 2:**
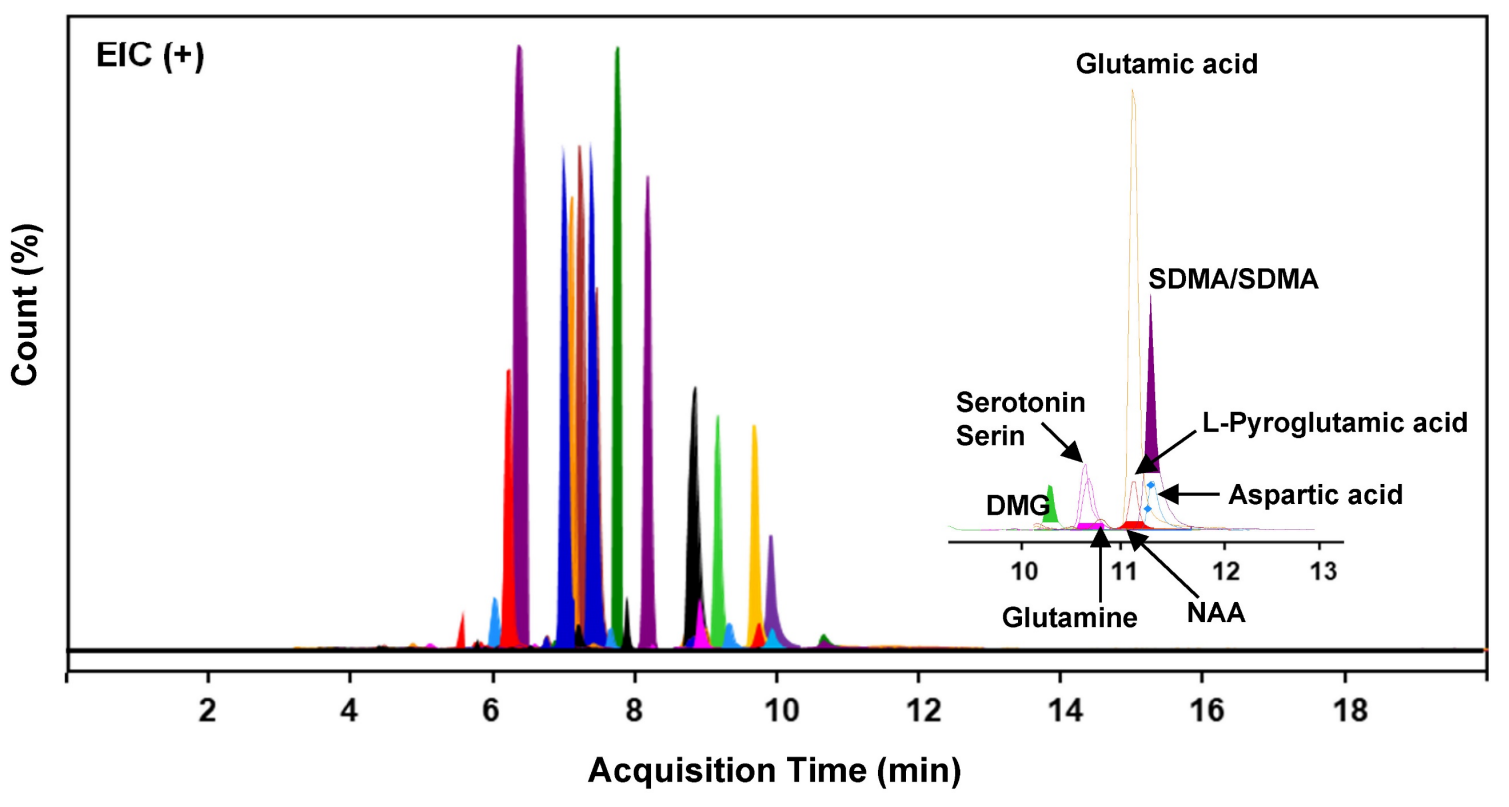
Extracted ion chromatograms (EIC) of 36 distinct compounds detected in SUEHP. DMG,* n*,*n*-dimethylglycine; NAA, *n*-acetyl-*L*-aspartic acid; SDMA, symmetric dimethylarginine.

The composition of primary metabolites in SUEHP was elucidated via UHPLC Q-TOF MS analysis, utilizing parameters such as relative retention time, m/z of precursor, and MS/MS fragmentation patterns. A comprehensive screening identified 1895 naked features, among which 503 metabolites were putatively matched with reference peaks corresponding to known primary and secondary metabolites. From this subset, 36 significant primary metabolites were distinctly characterized, including seven pivotal compounds: symmetric/asymmetric dimethylarginine (SDMA/ADMA), aspartic acid, glutamic acid, glutamine, *L*-pyroglutamic acid, *n*,*n*-dimethylglycine, and *n*-acetyl-L-aspartic acid (NAA) (**Fig. 2** and **Supplementary Table S1**). These compounds indicate potential interactions with specific biological targets, thereby suggesting pathways through which SUEHP may manifest its therapeutic effects.

**Figure 3:**
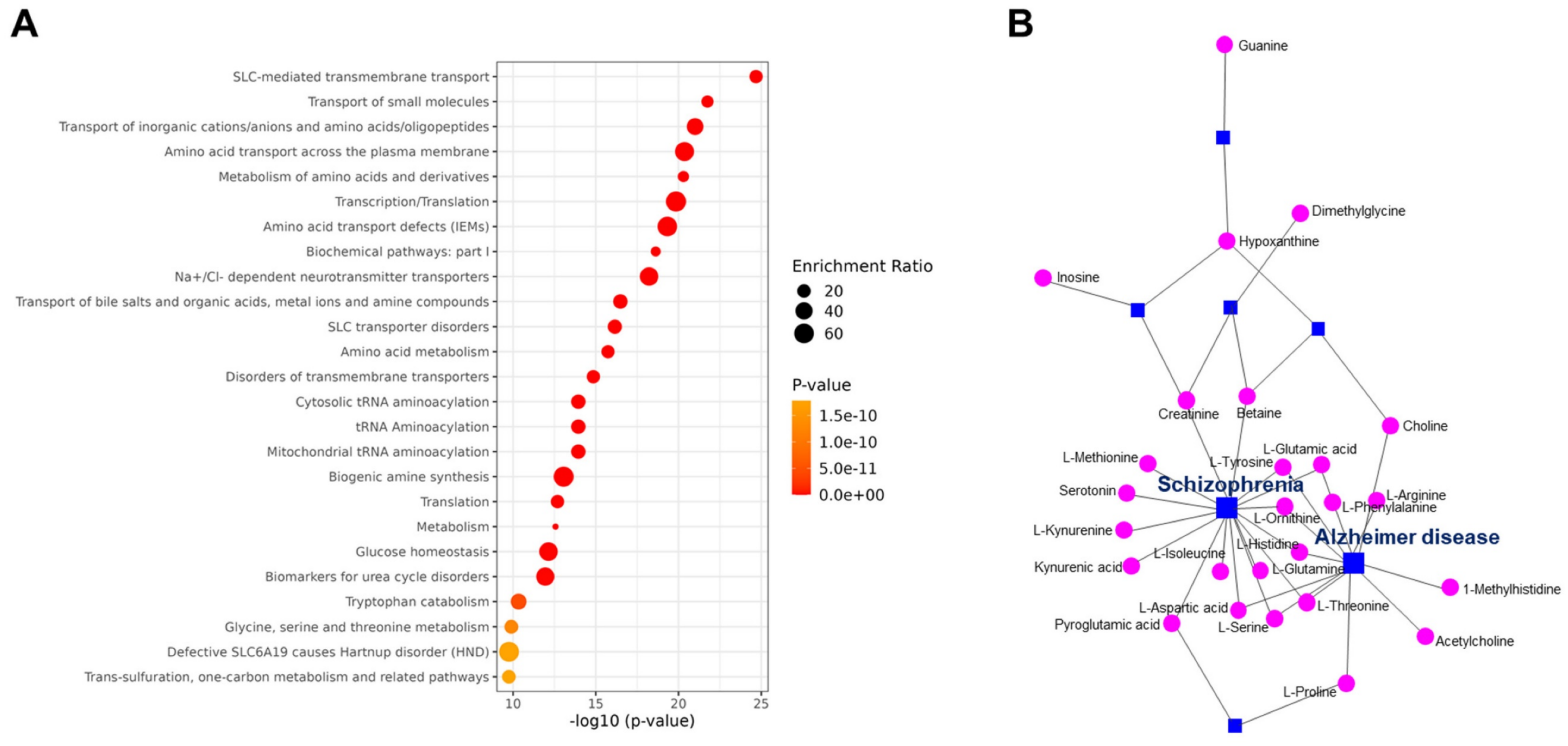
(A) Pathway enrichment analysis of differential metabolites. The color and size of each point indicate the *p*-value significance and the number of differentially enriched metabolites, respectively. (B) Metabolites-disease network analysis of the differentially expressed metabolite. Each line indicates the direct connection between metabolites (pink dots) and their associated diseases (blue squares).

Enrichment analysis of the metabolites identified by LC-MS revealed that the differentially expressed SUEHP components impact transmembrane transport and metabolism of small molecules (**Fig. 3A**). Subsequent network analysis revealed the connections between metabolites and several disease states highlighting the potential mechanistic links. Notable associations included the metabolites linked to neurodegenerative diseases including Alzheimer disease and schizophrenia, which underscores the pharmacological relevance of SUEHP components in influencing disease processes (**Fig. 3B**).

### Antioxidant activities of SUEHP

**Figure 4:**
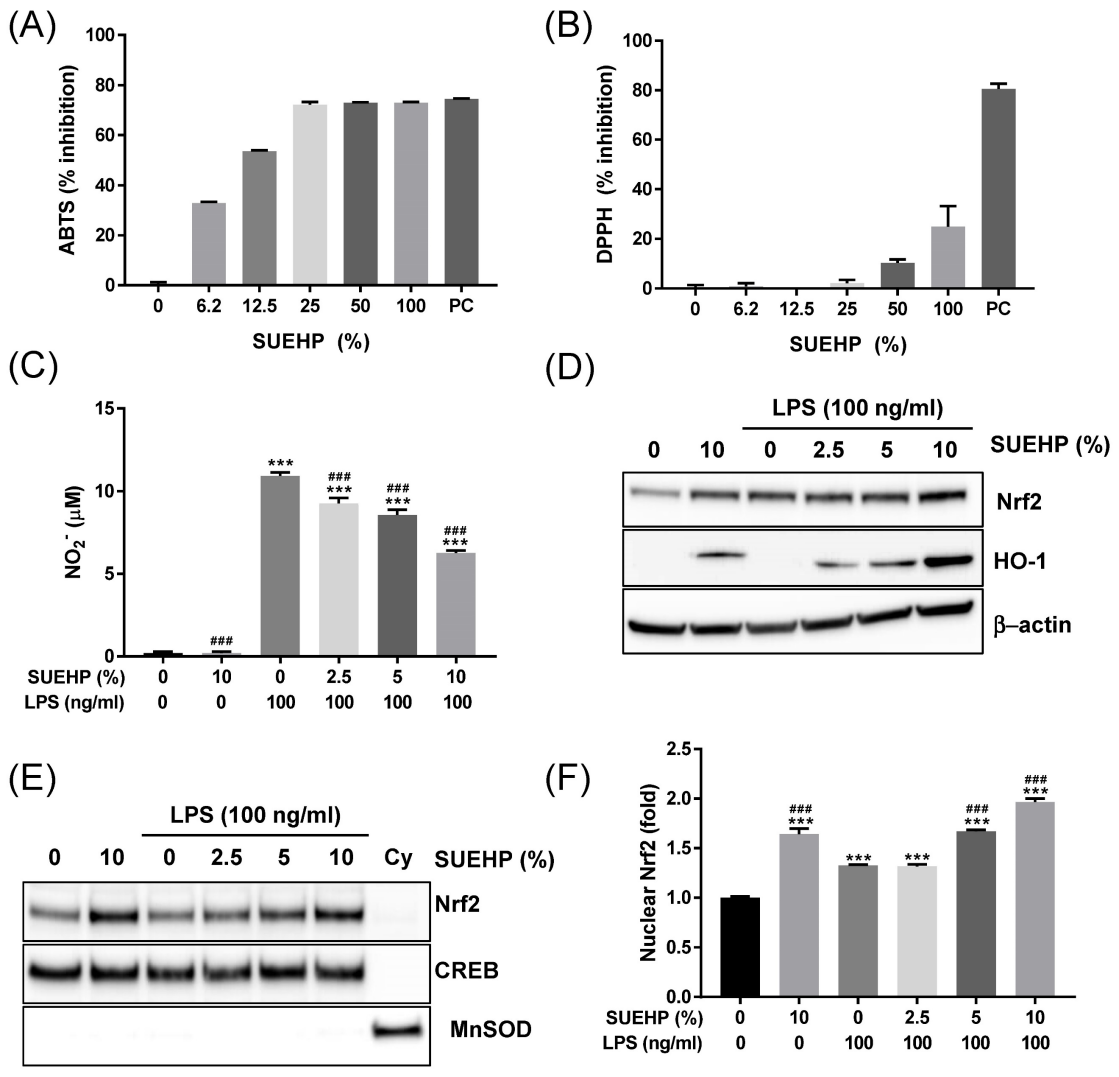
Antioxidant activity of SUEHP. The cationic and anionic free radical scavenging activities of SUEHP were determined using ABTS (A) and DPPH (B) assays, respectively. Nitric oxide (NO) production from BV2 microglial cells stimulated with 100 ng/ml LPS were determined in the presence and absence of SUEHP pretreatment (C). Changes of Nrf2/HO-1 antioxidant signaling were determined by western blot using the whole cell lysates(D). β-actin was included as a loading control. Nuclear translocation of activated Nrf2 proteins was determined by western blot (E) using the nuclear or cytoplasmic fractions. CREB and MnSOD were included as nuclear and cytoplasmic protein markers to verify the efficiency of subcellular fraction. Nuclear Nrf2 translocation was also quantified by ELISA using the nuclear lysates (F). Full length gel images for western blots are presented in **Supplementary Fig. S2**. Data were presented as means ± S.D. n = 2 per group except for Fig. 4C (n = 3). ^***^, *p*<0.001 *vs*. normal control cells; ^###^, *p*<0.001 *vs*. LPS only treated cells.

Dysregulated reactive oxygen species balance is known to be associated with various diseases, including cancer, inflammatory diseases^25^, diabetes^26^, neurodegenerative brain disease^27^, and pain^28^. Therefore, in the present study, we first investigate the antioxidant potential of SUEHP by performing cell-free- as well as in a cell culture system. First, ABTS and DPPH assays were applied to determine the activity of SUEHP scavenging cationic and anionic free radicals, respectively, in test tubes. As shown in **Fig. 4A**, SUEHP could scavenge ABTS+ radical in dose-dependent manners. The cationic radical scavenging activity of SUEHP at 25% (vol/vol) was comparable to 1 mM ascorbic acid, a positive control agent. IC_50_ value of ABTS for SUEHP was 10.3±0.2% vol of SUEHP. On the contrary, however, SUEHP did not efficiently remove anionic DPPH radicals, only showing 25.0±8.3% inhibition at the maximum concentration of SUEHP (100%, **Fig. 4B**). Ascorbic acid at 1 mM showed 80.5±2.2% inhibition of DPPH, which was similar to our in-house historical data.

Antioxidant potential of SUEHP was also evaluated in the BV2 murine microglial cell culture system. It was well known that the intracellular ROS level can be increased in BV2 cells when exposed to LPS^29^. So, we determined the intracellular nitric oxide (NO) levels in BV2 cells exposed to LPS in the presence and the absence of SUEHP. First, we determined the effect of SUEHP on cell viability in BV2 cells to avoid the influence of SUEHP cytotoxicity on cell-based *in vitro* assays. SUEHP up to 10% (vol/vol) did not show significant cytotoxicity in BV2 cells (**Supplementary Fig. S1.**). Therefore, we selected 10% SUEHP as the maximum concentration for subsequent *in vitro* assays with BV2 cells. Treatment of BV2 cells with LPS alone increased significantly intracellular NO. However, SUEHP pretreatment could inhibit NO production in a dose-dependent manner from the BV2 cells exposed to LPS, and SUEHP at 10% (vol/vol) could reduce the intracellular NO level by 43% compared with LPS only-treated cells (**Fig. 4C**). Interestingly, in the absence of LPS challenge, SUEHP treatment only could slightly but significantly induce NO production.

In our previous report, Nrf2/HO-1-mediated antioxidant pathway could be upregulated in LPS-treated BV2 cells as a cellular defense mechanism against oxidated stress^30^. To further elucidate the antioxidant activity of SUEHP, we investigated the intracellular Nrf2/HO-1 signaling in BV2 cells exposed to LPS in the presence and absence of SUEHP. When compared with the normal control cells, BV2 cells treated with LPS remarkably increase the intracellular Nrf2 expression and pretreatment of SUEHP could further enhance the expression of Nrf2 and HO-1 antioxidant proteins (**Fig. 4D**). In stressful condition, the Nrf2-Kelch-like ECH-associated protein 1 (Keap1) complex was disrupted and free Nrf2 protein translocates to the nucleus, where it binds to antioxidant response element (ARE) and induces transcription of Nrf2-regulated antioxidant genes like HO-1, glutathione S-transferase (GST) and NAD(PH) quinone dehydrogenase 1 (NQO-1) 31. Our western blot (**Fig. 4E**) and ELISA (**Fig. 4F**) assays demonstrated that LPS treatment increased the nuclear Nrf2 proteins compared with the normal control cells, which was enhanced by SUEHP pretreatment in a dose dependent manner.

### Anti-inflammatory activities of SUEHP

**Figure 5:**
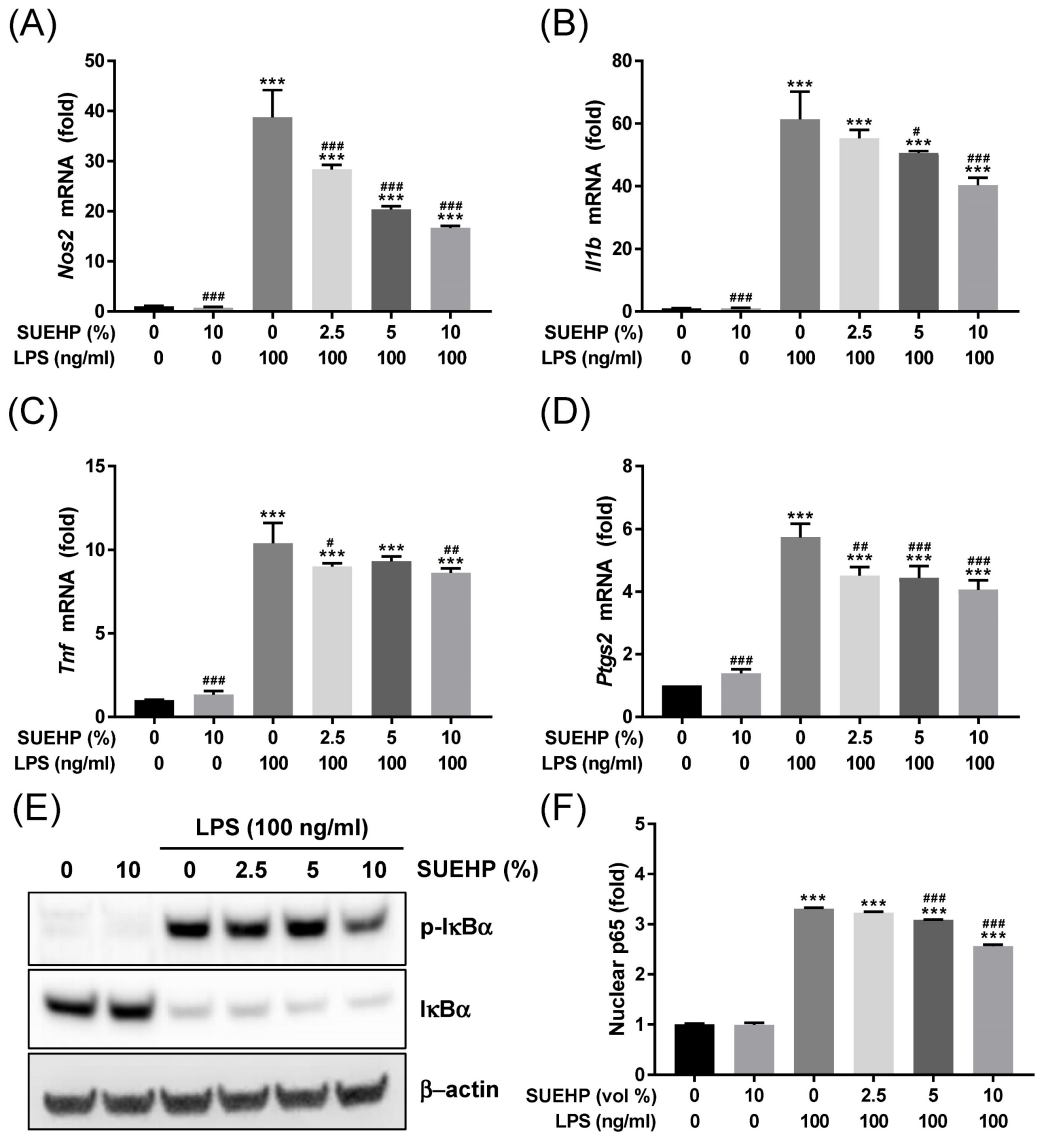
Anti-inflammatory activity of SUEHP. Expression of *Nos2* (A) and inflammatory cytokine (B-D) genes in the BV2 cells treated with 100 ng/ml LPS were determined by qPCR in the presence and absence of SUEHP pretreatment. Relative expression of specific genes was compared with that of normal control cells. Changes of NFκB signaling pathway by SUEHP pretreatment was determined by the western blot in the BV2 cells exposed to 100 ng/ml LPS (B). Nuclear translocation of activated NFκB p65 protein were also determined by the ELISA method. Full length gel images for western blots are presented in **Supplementary Fig. S2**. Data were presented as means ± S.D. n = 3 per group. ^***^, *p*<0.001 *vs*. normal control cells; ^#^, *p*<0.05, ^##^, *p*<0.01, ^###^, *p*<0.001 *vs*. LPS only treated cells.

Next, we further investigated whether SUEHP can inhibit the LPS-mediated inflammation in the BV2 microglial cells by qPCR determining of intracellular expression of inflammatory cytokine genes. LPS treatment could increase the expression of *Nos2* (**Fig. 5A**), *Il1b* (**Fig. 5B**), *Tnf* (**Fig. 5C**), and *Ptgs2* (**Fig. 5D**) genes. However, pretreatment of SUEHP successfully alleviate the LPS-mediated upregulation of inflammatory cytokine genes in dose dependent manners. Similar to the results from the antioxidant assays, SUEHP only treatment could slightly but significantly increase the expression of cytokine genes in the absence of LPS. As the NFκB signaling pathway is well established in LPS-mediated inflammation, we determined the NFκB signaling activation in LPS-treated BV2 cells in the presence of SUEHP by western blotting (**Fig. 5E**) and ELISA (**Fig. 5F**) assays. Following LPS treatment, phosphorylation of IκBα at serine 32 significantly increased resulting degradation of IκBα (**Fig. 5E**) and unleashed NFκB p65 protein from IκBα translocated to the nucleus (**Fig. 5F**). SUEHP pretreatment, however, could alleviate LPS-mediated inflammatory response in a dose dependent manner, by inhibiting phosphorylation of IκBα and therefore, decreasing nuclear translocation of NFκB p65 protein.

### Biochemical target screening of SUEHP

To obtain the clue of potential protein targets of SUEHP, a pan-biochemical screening test was performed at a fixed SUEHP concentration (1%) as described earlier. Among 87 biochemical assays, ionotropic (*N-methyl-D-aspartate* (NMDA), a-amino-3-hydroxy-5-methyl-4-isoxazolepropionic acid (AMPA), Kainate) and metanotropic (mGlu5) glutamate receptors showed high responses to SUEHP followed by insulin and glycine receptors, COX-2 enzyme, ATPase, and glucocorticoid receptor (**Table 3**).

As COX-2 is one of the key enzymes responsible for inflammation, the regulation of COX-2 and its isoenzyme COX-1 by SUEHP was further confirmed by a biochemical assay. Specific inhibitors for COX-1 (SC-560) and COX-2 (DuP-697) were also included in the assay to confirm the validity of assay system as well as to compare them with the inhibitor potential of SUEHP. As shown in Fig.4, SUEHP could inhibit COX-1 and COX-2 enzymes in dose dependent manners and showed 25% and 35% inhibition at maximum dose of SUEHP against COX-1 and COX-2, respectively (**Fig. 4**). Specific inhibitors for COX-1 (SC-560) and COX-2 (DuP-697) exhibited 88% and 98% inhibition, respectively, which were similar to the values suggested in the manufacture's instruction.

## Discussion

PA has been widely used in TKM since the principle of PA was officially introduced in 1967 by Sang-Cheon Nam who injected the extracts of Astragali Radix, Ginseng Radix, Ziziphi Semen, and Cervi Parvum Cornu on the acupoints based on meridian theory^32^. PA in TKM differs from aqua-acupuncture in traditional Chinese medicine (TCM) in that PA in TKM is prescribed based on TKM theory including meridian system and Qi/Flavor theory, and does not combine PA with Western medicine^2^. In addition, the qua-acupuncture in TCM administers drugs through intramuscular or even intravascular injections as well as acupoints^33^, which means that TKM PA has been developed independently of aqua-acupuncture in TCM.

A recent bibliometric analysis on the PA demonstrated that clinical studies with highest level of evidence like pragmatic clinical trials or randomized controlled trials were recently performed in PA fields 2. However, the information of chemical compositions comprising PA is limited. Since biological or pharmaceutical activities of drugs depend on their chemical entities, a comprehensive analysis of chemical composition of PA is critical to understand its mechanism of action. The present study aimed to identify chemical compositions of SUEHP using GC-MS and LC-MS methods, and to evaluate its biological activities using cell-free as well as cell culture systems using BV2 murine microglial cells.

**Figure 6:**
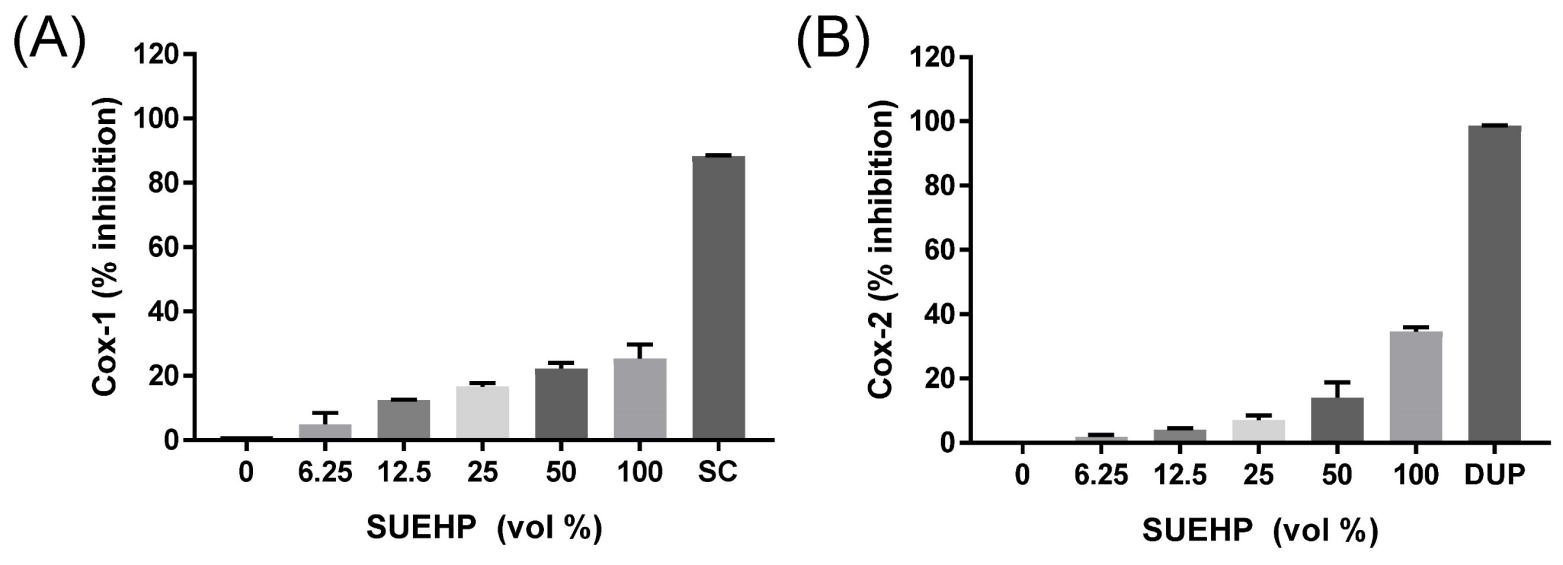
Inhibition of COX enzyme activity by SUEHP. Activities of ovine COX-1 and recombinant human COX-2 isozymes were biochemically determined in the presence of serially diluted SUEHP as described earlier. SC and DUP represent SC-560 and DuP-697 that were included as COX-1 and COX-2 specific inhibitors, respectively. Data were presented as means ± S.D. n = 2 per group.

As determined by GC-MS of SUEHP, a diverse hydrophobic volatile hydrocarbons were identified in SUEHP, which may be due to the SUEHP manufacturing process which includes evaporation of herbal extract solution except for Cervi Parvum Cornu. As a most abundant compound identified in GS-MS analysis of all three solvent fractions of SUEHP, 9-octadecenamide (or oleamide) comprises a family of amidated lipids found in cerebrospinal fluid and plasma of mammals including human^34^. Like hexadecanamide that was also identified in all three solvent fractions of SUEHP, 9-octadecenamide acts as a ligand of PPARα in hippocampal nuclei and controls hippocampal plasticity *via* transcriptional activation of CREB^35^. In addition, 9-octadecenamide regulates feeding and sexual behavior^36^, modulates memory function^37^, and exerts hypnotic, analgesic and anxiolytic action through regulating γ-aminobutyric acid A (GABA_A_) receptor, serotonin (5HT) receptor, voltage-gated sodium channels, and cannabinoid receptor 1 (CB1)^38^. *Codium fragile* extracts which contain hexadecanamide and 9-octadecenamide as major components could improve pulmonary dysfunction of mice that were chronically exposed fine dust through activating antioxidant system and attenuating TLR4-mediated inflammation and TGF-β-mediated pulmonary fibrosis in lung tissues^39^. Hexadecanamide and 9-octadecenamide was also found in the extract of deer velvet antler extracts that is one of components of SUEHP and was known to improve arthritis by anti-inflammatory potential^40^. Oral administration or topical application of Gano oil that is rich in fatty acids like hexadecanamide and 9-octadecenamide could relieve inflammatory pain in multiple animal models by inhibition of canonical NFκB-mediated inflammatory signaling and COX enzyme activity as well as by antioxidant activity of scavenging free radicals^41^, which was similar with our anti-inflammatory (Fig. 5 and Fig. 6) and antioxidant (Fig. 4) activities of SUEHP.

9-octadecenoic acid or elaidic acid that was only identified in DW-ACN fraction of SUEHP was known to suppress tumor growth by boosting major histocompatibility complex class I (MHC-1)-mediated tumoral antigen presentation *via* acyl-coenzyme A (CoA) synthetase long-chain family 5^42^. Its cis-isomeric form, cis-11-octadecenoic acid or cis-vaccenic acid which was identified in both n-hexane and DCM fractions of SUEHP, was previously found in the lysate of an immortalized cell line derived from the striatum (X61) and was capable of increasing dopamine expression in immortalized mouse mesencephalic cell line (MN9D) revealing a therapeutic potential of PD that is related with dopaminergic cell loss^43^.

As a volatile fatty alcohol found in vegetable or animal foods, 1-octanol was identified in all 3 different solvent extraction used in GC-MS analysis. In previous studies, it could reduce essential tremor in experimental rat models by blocking abnormal activation of T-type calcium channel (Ca_v_3)^44^, ^45^. A small cohort pilot clinical study showed that an oral intake of single low dose of 1-octanol could reduce tremor amplitude lasting for 90 min without any significant adverse effects^46^. 1-octanol identified as one of major active components of frankincense (also known as olibanum) oil also showed anti-inflammatory and analgesic effects in mice by inhibiting nociceptive stimulus-induced inflammatory infiltrates and COX-2 overexpression^47^. In our study, SUEHP was able to reduce the gene expression of inflammatory cytokine like IL-1β, TNFα, and COX-2 in LPS-stimulated BV2 murine microglia cells by inhibiting a NFκB signaling pathway (Fig. 4). In our pan-biochemical assay, SUEHP could modulate COX-2 enzyme and glucocorticoid receptor (**Table 3**), which are pharmaceutical targets of anti-inflammatory drugs^48^. In addition, SUEHP was able to inhibit COX-1 and COX-2 enzyme activity in a cell-free biochemical assays (Fig. 6), which are related with anti-inflammatory effects of SUEHP. Olibanum is one of medicinal herbs comprising SUEHP and therefore, it may in partial contribute to anti-inflammatory potential of SUEHP.

As an a high-melting point amide non-ionic surfactant found in many animals and plants, hexadecanamide (a common name palmitamide) was known to exert anti-inflammatory activity by activating peroxisome proliferator-activated receptor alpha (PPARα)-sirtuin 1 (SIRT1) signaling^48^. Recent animal study showed that hexadecanamide exists as an endogenous ligand of PPARα in hippocampal nuclei, and oral administration of hexadecanamide could BDNF expression in hippocampus and improve the cognitive behavior through PPARα signaling in a 5XFAD transgenic Alzheimer moue model^49^.

Eicosane is a C20-long chain hydrocarbon that was detected in hydrophobic solvent like n-hexane and DCM in the present study. Topical application of eicosane could accelerate wound healing in a diabetic rat model via their moderate potential of antioxidant activity like robust free radical scavenging, hydroxyproline and glutathione action, and via elevated epithelialization, fibroblast movement, polymorphonuclear leukocyte migration, and collagen synthesis in the wound area by modulating matrix metalloproteinase (MMP) activities^50^. In a neuronal system, excessive inflammatory activation by glial cells leads to exaggerated release of glutamate from glial cells to extracellular space^51^, and excitotoxic concentration of extracellular glutamate induces ROS over-production from mitochondria in neuronal cells following NMDA receptor activation and intracellular Ca^2+^ entry^52^. Antioxidant and anti-inflammatory properties of eicosane protected R28 retinal precursor cells *in vitro* as well as retinal ganglion cells (RGC) from glutamate-NMDA-mediated excitotoxicity in mouse glaucoma model through L-arginine and/or L-carnitine metabolism^53^. Eicosane also showed the antinociceptive and anti-inflammatory potential without adverse effect through *in silico* prediction of biological activity and toxicity^54^.

Besides, dotriacontane^55^ 3,5-di-tert-butylphenol^56^ showed anti-inflammatory activity by reducing prostaglandins through inhibition of COX enzyme activity. Some minor components like verticiol^57^, cis-9-hexadecenal^58^, octadecane^59^ might contribute the antioxidant and anti-inflammatory effects of SUEHP.

The enrichment analysis suggested the potential for SUEHP components to modulate pathways related to transmembrane transporter and small molecule metabolism. This suggests that SUEHP may exert its therapeutic effects through the regulation of cellular transport mechanisms and the metabolism of biologically active molecules. Such effects on transmembrane transport could be pivotal, potentially facilitating the enhanced cellular uptake or efflux of therapeutic compounds, thereby influencing cellular homeostasis and response to treatment. Furthermore, the network analysis showed associations between SUEHP components and disease, particularly highlighting links with neurodegenerative diseases. The identification of components associated with diseases like Alzheimer's disease and schizophrenia may potential pathways through which SUEHP could modulate disease progression or symptomatology.

## Conclusion

In conclusion, the present study demonstrated the anti-inflammatory and antioxidant activities of SUEHP using *in vitro* assay systems and identified its chemical components through comprehensive GC-MS and LC-MS analyses that may contribute its biological activities. Comprehensive chemical analysis of PA may guide us to understand the underlying mechanism of pharmaceutical effects of PA in treatment of diseases or management of disease-related symptoms. The limitation of the present study was the fact that the biological activities of SUEHP was only evaluated using *in vitro* assay systems and therefore, the pharmacological values of SUEHP should be further validated in suitable preclinical disease models that are associated with inflammation and oxidative stress.

## Supplementary Material

Supplementary figures and table.

## Figures and Tables

**Table 1:** Sequence of gene-specific primers for qPCR.

Target	Forward (5′ → 3′)	Reverse (5′ → 3′)	Ref. sequence
Il1b	GCTGAAAGCTCTCCACCTCA	AGGCCACAGGTATTTTGTCG	NM_008361.4
Nos2	GAATCTTGGAGCGAGTTGTGGA	GTGAGGGCTTGGCTGAGTGAG	NM_010927
Ptgs2	TTCAACACACTCTATCACTGGC	AGAAGCGTTTGCGGTACTCAT	NM_011198.4
Tnf	AGACCCTCACACTCAGATCATCTTC	CCACTTGGTGGTTTGCTACGA	NM_013693
Gapdh	AAGGTGGTGAAGCAGGCAT	GGTCCAGGGTTTCTTACTCCT	NM_001001303

**Table 2:** The qualitative analysis results of SUEHP extracted using n-hexane, dichloromethane, or deionized water-acetonitrile mixture by GC-MS.

Solvent	Peak	R_t_^*^ (min)	Compounds	Mol. Formula	%
n-Hexane	1	8.77	4-Methyldecane	C11H24	0.54
	2	9.33/10.07	3,7-Dimethyldecane	C12H26	1.85
	3	9.42	3,8-Dimethylundecane	C13H28	0.59
	4	9.56	1-Octanol	C8H18O	0.50
	5	11.66	Dodecane	C12H26	0.64
	6	12.57	5-Methyltetradecane	C15H32	0.86
	7	12.76/13.42	Octadecane	C18H38	2.50
	8	14.50	Tetradecane	C14H30	0.52
	9	14.65	3,8-Dimethyldecane	C12H26	0.68
	10	15.65/18.18	Eicosane	C20H42	5.42
	11	15.87	3,5-Di-tert-butylphenol	C14H22O	0.85
	12	16.22	8-Methylheptadecane	C18H38	0.88
	13	20.29	2-ethyl 2-methyl tridecanol	C16H34O	1.28
	14	21.72	Cembrene	C20H32	0.50
	15	22.61	cis-11-Octadecenoic acid	C18H34O2	1.05
	16	23.00	Hexadecanamide	C16H33NO	1.26
	17	24.60	9-Octadecenamide	C18H35NO	25.88
	18	24.81	Dodecanamide	C12H25NO	1.41
	19	26.07	Dotriacontane	C32H66	2.18
	20	26.21	Oleyl alcohol	C18H36O	1.23
DCM^*^	1	8.77	5-Ethyl-2-methylheptane	C10H22	0.68
	2	9.33/10.07	3,7-Dimethyldecane	C12H26	2.50
	3	9.56	1-Octanol	C8H18O	1.05
	4	11.65	Dodecane	C12H26	1.37
	5	11.84	2,5-Dimethylundecane	C13H28	0.56
	6	12.76	Octadecane	C18H38	2.11
	7	15.65/18.19	Eicosane	C20H42	2.94
	8	15.87	3,5-Di-tert-butylphenol	C14H22O	1.79
	9	20.37	Dotriacontane	C32H66	0.80
	10	21.72	Cembrene	C20H32	0.71
	11	22.61	cis-11-Octadecenoic acid	C18H34O2	3.14
	12	23.00	Hexadecanamide	C16H33NO	1.20
	13	24.60	9-Octadecenamide	C18H35NO	29.14
	14	26.21	Oleyl alcohol	C18H36O	1.80
DW-ACN^*^	1	7.82	Hexanoic acid	C6H12O2	2.06
	2	9.56	1-Octanol	C8H18O	1.69
	3	20.35	*cis*-11-Hexadecenal	C16H30O	2.58
	4	21.33	*cis*-9-Hexadecenal	C16H30O	0.69
	5	21.72	Verticiol	C20H34O	1.14
	6	22.61	9-Octadecenoic acid	C18H34O2	17.92
	7	23.00	Hexadecanamide	C16H33NO	2.36
	8	24.42	Heptaethylene glycol	C14H30O8	1.39
	9	24.60	9-Octadecenamide	C18H35NO	31.07
	10	25.07	Oleoyl chloride	C18H33ClO	2.50
	11	26.21	Oleyl alcohol	C18H36O	1.85
	12	26.67	Hexaethylene glycol	C12H26O7	1.22
	13	30.97	Cholesterol	C27H46O	2.28
	14	31.41	Undecaethylene glycol	C22H46O12	1.36

^*^R_t_, retention time; DCM, dichloromethane; DW-ACN, 1:1 mix of deionized water and acetonitrile. Relative peak area (%) of the individual components was calculated based on total GC peak areas.

**Table 3:** Summary of pan-biochemical assay.

Assay No	Assay Name	Species	SUEHP (%)	Response (%)
232910	Glutamate, NMDA, glycine	Rat	1	105
232810	Glutamate, NMDA, agonism	Rat	1	101
232600	Glutamate, AMPA	Rat	1	99
232710	Glutamate, Kainate	Rat	1	99
237000	Glutamate, metabotropic, mGlu5	Human	1	99
174990	Protein tyrosine kinase, insulin receptor	Human	1	82
239000	Glycine, strychnine-sensitive	Rat	1	75
118030	Cyclooxygenase COX-2	Human	1	58
107710	ATPase, Na^+^/K^+^, heart, pig	Pig	1	56
232030	Glucocorticoid	Human	1	53

The significant responses showing ≥50% inhibition or stimulation for biochemical assays were noted.

## Abbreviations

5HT: serotonin
ABTS: 2,2′-azino-bis[3-ethylbenzothiazoline-6-sulfonic acid
ADMA: asymmetric dimethylarginine
ARE: antioxidant response element
BCA: bicinchoninic acid assay
BDNF: brain-derived neurotrophic factor
Ca_v_3: T-type calcium channel
CB1: cannabinoid receptor 1
CoA: acyl-coenzyme A
COX: cyclooxygenase
CREB: cAMP response element-binding protein
DCM: dichloromethane
DMG: n,n-dimethylglycine
DPPH: 1,1-diphenyl-2-picrylhydrazyl
DW-ACN: deionized water-acetonitrile
EDTA: ethylene-diamine-tetraacetic acid
EIC: extracted ion chromatograms
FBS: fetal bovine serum
GABA_A_: γ-aminobutyric acid A
Gapdh: glyceraldehyde 3-phosphate dehydrogenase
GC-MS: gas chromatography-mass spectrometry
GST: glutathione S-transferase
HO-1: heme oxygenase-1
HRP: horseradish peroxidase
IκBα: nuclear factor of kappa light polypeptide gene enhancer in B-cell inhibitor alpha
Il1b: interleukin 1 beta
Keap1: Kelch-like ECH-associated protein 1
LC-MS: liquid chromatography-mass spectrometry
LPS: lipopolysaccharide
MHC-1: major histocompatibility complex class I
MMP: matrix metalloproteinase
MnSOD: manganese superoxide dismutase
NAA: n-acetyl-L-aspartic acid
NFκB: nuclear factor kappa-light-chain-enhancer of activated B cells
NO: nitric oxide
Nos2: nitric oxide synthase 2
NQO-1: NAD(PH) quinone dehydrogenase 1
Nrf2: erythroid 2-related factor 2
OD: optical density
PA: pharmacopuncture
PBS: phosphate-buffered saline
PD: Parkinson's disease
PPARα: peroxisome proliferator-activated receptor alpha
Ptgs2: prostaglandin-endoperoxide synthase 2
qPCR: quantitative polymerase chain reaction
Q-TOF-MS: quadrupole time-of-flight mass spectrometry
RGC: retinal ganglion cells
SDMA: symmetric dimethylarginine
SIRT1: sirtuin 1
SUEHP: SU-Eohyeol PA
TA: transcription factor
TBS-T: Tris-buffered saline containing 0.1% Tween 20
TCM: traditional Chinese medicine
TKM: traditional Korean medicine
Tnf: tumor necrosis factor
UHPLC: ultra-high performance liquid chromatography
