# Supplementary material for: Chemical Analysis of SU-Eohyeol Pharmacopuncture and Its In Vitro Biological Activities
Source: Int J Med Sci. 2024 Oct 3;21(13):2562–77. doi: 10.7150/ijms.100083 (PMC11492884; doi:10.7150/ijms.100083)

Table S1. List of the identified 36 metabolites in SUEHP.

**Metabolites**	**Formula**	**Theoretical M/Z**	**Adduct**	**RT**	**Observed M/Z**	**Accuracy**	**Fragment ions***
1-Methylguanosine	C11H15N5O5	298.1151	[M+H]+	7.56	298.1146	1.61	166.0726, 149.0450
3-Hydroxyproline	C5H9NO3	132.066	[M+H]+	10.11	132.0663	1.97	114.0657, 86.0602, 68.0499
3-Methylhistidine	C7H11N3O2	170.0929	[M+H]+	11.09	170.0927	1.29	153.0650, 109.0761, 96.0680, 83.0580
4-Hydroxyquinoline	C9H7NO	146.0606	[M+H]+	4.45	146.0600	3.83	128.0520, 118.0650, 91.0546, 77.0400
5-Hydroxytryptophan	C11H12N2O3	221.0926	[M+H]+	8.97	221.0922	1.76	186.0550, 162.0552, 134.0605
Acetylcholine	C7H16NO2	146.1181	[M]+	10.52	146.1183	1.57	87.0442, 60.0812
Adenine	C5H5N5	136.0623	[M+H]+	6.93	136.0620	1.91	119.0353, 109.0510, 94.0401, 67.0291
ADMA	C8H18N4O2	203.1508	[M+H]+	12.02	203.1503	2.56	158.1291, 116.0702, 88.0873, 70.0653
Arginine	C6H14N4O2	175.1195	[M+H]+	12.07	175.1197	1.03	158.0924, 130.0970, 116.0709, 60.0570
Aspartic acid	C4H7NO4	134.0453	[M+H]+	11.35	134.0450	2.39	88.0394, 74.0237, 43.017
Betaine	C5H11NO2	118.0868	[M+H]+	8.92	118.0864	3.81	59.0733, 58.0690
Choline	C5H14NO	104.1075	[M]+	7.97	104.1078	2.88	60.0814, 58.0660
Creatinine	C4H7N3O	114.0687	[M+H]+	7.31	114.0683	3.59	86.0716, 72.0448, 57.0448
DL-Norleucine	C6H13NO2	132.1024	[M+H]+	8.27	132.1029	3.78	132.1021, 86.0968, 69.0720, 55.0210
Glutamic acid	C5H9NO4	148.061	[M+H]+	11.14	148.0607	1.82	130.0497, 102.0552, 84.0445, 56.0498
Glutamine	C5H10N2O3	147.0769	[M+H]+	10.53	147.0772	1.77	102.0580, 84.0444, 56.0498
Guanine	C5H5N5O	152.0572	[M+H]+	8.14	152.0575	1.97	135.0310, 110.0349, 55.0289
Histidine	C6H9N3O2	156.0773	[M+H]+	10.78	156.0781	5.25	110.0712, 93.0440, 83.0607, 68.0490
Hypoxanthine	C5H4N4O	137.0463	[M+H]+	7.12	137.0469	4.67	119.0351, 110.0346, 94.0398, 82.0408
Inosine	C10H12N4O5	291.0702	[M+Na]+	8.01	291.0704	0.69	137.0470, 119.0350, 55.0330
Isoleucine	C6H13NO2	132.1024	[M+H]+	8.55	132.1024	0.3	86.0966, 69.0720, 57.0579, 56.0540
Kynurenic acid	C10H7NO3	190.0504	[M+H]+	7.38	190.0497	3.95	144.0443, 116.0499
L-Kynurenine	C10H12N2O3	209.0926	[M+H]+	8.19	209.093	1.82	174.0555, 146.0608, 118.0658, 74.0244

L-Pyroglutamic acid	C5H7NO3	130.0504	[M+H]+	11.15	130.0497	5.07	84.0445, 65.0386, 56.0493
Methionine	C5H11NO2S	150.0588	[M+H]+	8.76	150.0586	1.6	149.0270, 102.0543, 74.0240, 56.0499
N,N-Dimethylglycine	C4H9NO2	104.0711	[M+H]+	9.27	104.0713	1.63	58.0656
N-Acetyl-*DL*-aspartic acid	C6H9NO5	176.0559	[M+H]+	10.81	176.0552	4.03	134.0437, 116.0344, 88.0395, 74.0233
Nicotinic acid	C6H5NO2	124.0398	[M+H]+	8.27	124.0398	0.24	116.0712, 70.0655
Ornithine	C5H12N2O2	133.0977	[M+H]+	12.19	133.0978	0.6	120.081
Phenylalanine	C9H11NO2	166.0868	[M+H]+	8.14	166.0867	0.72	70.0655
Proline	C5H9NO2	116.0711	[M+H]+	9.35	116.0717	4.74	172.1076, 116.0708, 88.0873, 70.0655
SDMA	C8H18N4O2	203.1508	[M+H]+	11.32	203.1507	0.54	88.0384, 70.0300, 60.0450
Serine	C3H7NO3	106.0504	[M+H]+	10.69	106.0499	5.09	142.0651, 132.0830, 115.0540, 105.0700
Serotonin	C10H12N2O	177.1028	[M+H]+	10.74	177.1023	2.94	102.0543, 74.0608, 56.0501
Threonine	C4H9NO3	120.066	[M+H]+	10.26	120.0656	3.41	136.0760, 119.0520, 91.0546, 77.0390
Tyrosine	C9H11NO3	182.0817	[M+H]+	9.09	182.0815	1.04	166.0726, 149.0450

*Product ions has been verified by MoNA (MassBank of North America) database.

Figure S1. Toxicity of SUEHP in BV2 mouse microglial cells. Relative cell viability was determined via a WST-8 assay after treatment with SUEHP for 24 h. n = 3 per group. $^{*}p<0.05$, $^{**}p<0.01$, and $^{***}p<0.001$ *vs*. normal control cells.

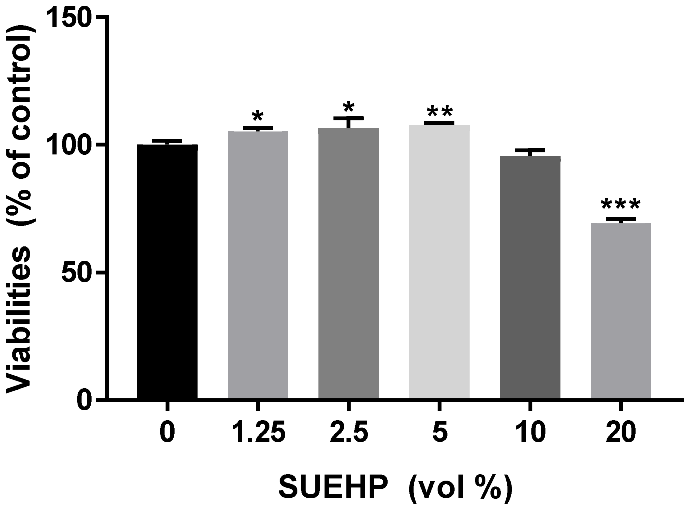

Figure S2. Uncropped membrane images for western blot analyses.

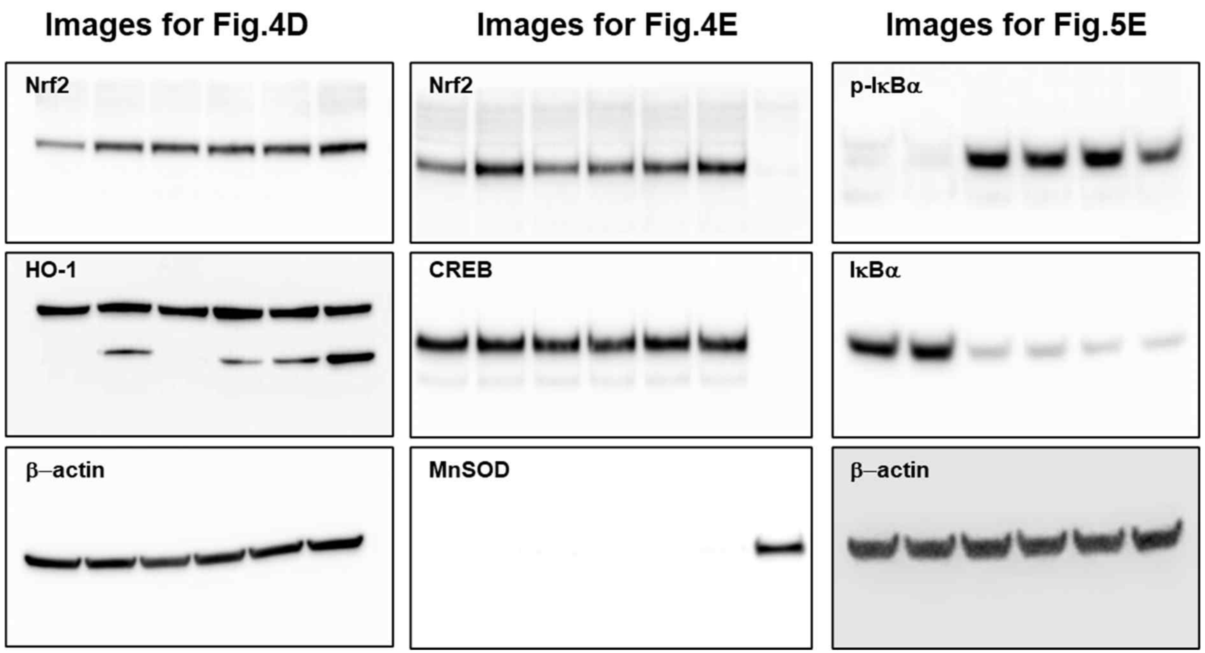

Supplement: Supplementary file 1 — Supplementary figures and table. [file ijmsv21p2562s1.pdf]
